# Reduced SULT2B1b expression alleviates ox-LDL-induced inflammation by upregulating miR-148-3P via inhibiting the IKKβ/NF-κB pathway in macrophages

**DOI:** 10.18632/aging.202273

**Published:** 2021-01-10

**Authors:** Mengzhuo Yin, Jianwen Lu, Zhongzhou Guo, Yanan Zhang, Jichen Liu, Tongwei Wu, Kai Guo, Tiantian Luo, Zhigang Guo

**Affiliations:** 1 Department of Cardiology, Huiqiao Medical Centre, Nanfang Hospital, Southern Medical University, Guangzhou 510515, People’s Republic of China; 2 Department of Endocrinology, Guangzhou First People’s Hospital, School of Medicine, South China University of Technology, Guangzhou 510180, People’s Republic of China

**Keywords:** sulfate transferases 2B1b, miR-148a-3p, inflammatory response, NF-κB, ox-LDL

## Abstract

Atherosclerosis is a lipid-driven chronic inflammatory disease in which lipid-laden macrophage foam cells lead to inflamed lesions in arteries. Previous studies have proven that sulfotransferase 2B1b (SULT2B1b) has several roles in the regulation of lipid metabolism and the inflammatory response. However, little is known about the functions of SULT2B1b in ox-LDL-induced inflammation in macrophages. In this study, after treatment with either ox-LDL alone or combined with transfection of siRNAs targeting SULT2B1b, IL-6, TNF-α, NF-κB, IKKβ and IκB mRNA and protein expression were determined in Raw264.7 cells by real-time PCR and Western blot, respectively. The proliferative capacity was determined by EdU staining and Cell Counting Kit-8. Our data demonstrated that SULT2B1b knockdown could reduce phosphorylated NF-κB levels and downregulate IKKβ protein levels. Additionally, IκB levels were increased and the proliferation of ox-LDL stimulated cells was inhibited after SULT2B1b silencing. Downregulation of SULT2B1b expression was found to upregulate miR-148a-3p expression by microarray assay, while IKKβ was a miR-148a-3p target gene. Our study suggests that SULT2B1b knockdown could promote miR148a-3p expression and inhibit activation of the IKKβ/NF-κB signalling pathway, which suppressed the inflammatory response in macrophages. Therefore, targeting the SULT2B1b gene might be potentially beneficial for atherosclerosis prevention by decreasing the inflammatory response.

## INTRODUCTION

Atherosclerosis (AS) is a chronic multifactorial disease with high incidence and mortality rates worldwide [1]. Currently, it is well-known that the pathophysiological mechanisms and aetiology of AS include the dysregulation of lipid metabolism, activation of inflammatory factors and functional activation of various cells in the blood vessels, such as lymphocytes and macrophages. Therefore, inflammation and lipid metabolism have aroused considerable attention in the field of AS research in recent years [2, 3]. Anti-inflammatory and lipid metabolism-regulating drugs, such as canakinumab and statins, have become important strategies for the treatment of AS [4]. Up to now, although many disease-causing factors have been shown to be associated with the development of AS, oxidized low-density lipoprotein (ox-LDL) remains a very important risk factor for inducing macrophages to express many inflammatory molecules, especially pro-inflammatory cytokines, which can further promote AS development [5–7]. AS is also considered to be a chronic inflammatory disorder, and growing evidence has shown the critical roles of inflammatory cytokines, such as interleukin-1β (IL-1β), IL-6 and tumour necrosis factor-α (TNF-α), which exhibit their unique functions during the progression of AS [8, 9]. In this respect, the prevention of inflammatory reaction in ox-LDL-exposed macrophages has attracted attention as a potential therapeutic target for the treatment of cardiovascular disease.

The cytoplasmic sulphate transferase (SULT) superfamily is a group of proteins that catalyse the addition of 3'-phosphoadenosine 5'-phosphate (PAP), which is transferred to compounds containing hydroxyl or amino groups to form sulphates or sulfamates, respectively [10]. The SULT2 family includes the SULT2A1 and SULT2B1 genes, which encode SULT2A1, SULT2B1a and SULT2B1b [11, 12]. Notably, SULT2B1b has been identified as a key enzyme in oxysterol sulphate synthesis and investigated for its role in the regulation of lipid metabolism [13], the inflammatory response [14] and cell proliferation [15]. Most importantly, our recent study demonstrated that SULT2B1b could promote cholesterol accumulation and induce inflammation by inhibiting LXR-β in lymphocytes in acute myocardial infarction patients with low LDL-C level [16]. Interestingly, this evidence shows that SULT2B1b is a potential target for controlling AS via its immunological functions that induce the inflammatory response. However, it is unclear whether SULT2B1b is involved in the induction of inflammation in macrophages after ox-LDL stimulation.

microRNAs (miRNAs), which are small noncoding RNAs, have been well-documented to serve as crucial role in biological processes [17]. The regulatory roles of miRNAs in various pathophysiological cellular effects and molecular signalling pathways in AS have also been demonstrated [18]. For example, increased expression of miR-125a-5p in THP-1 cells stimulated by ox-LDL may protect AS by regulating the expression of scavenger receptors [19]. miR-146a expression was significantly downregulated in THP-1 cells that were stimulated with ox-LDL and miR-146a could reduce the release of inflammatory factors, both of which affect the development of AS [18]. Furthermore, miR-155 reduced the uptake of lipoproteins and inhibited the inflammatory response in endothelial cells and macrophages that were treated with ox-LDL [20]. In addition, miR-342-5p promoted AS by enhancing the inflammatory response and inhibiting miR-155 expression [21]. These experimental data provide insights into the roles of key miRNAs in AS pathogenesis by regulating the inflammatory response. However, it is unknown whether SULT2B1b can induce alterations in miRNAs to modulate ox-LDL-stimulated inflammation in macrophages.

SULT2B1b and miRNAs play important roles in modulating lipid metabolism and inflammatory response in macrophages after stimulation with ox-LDL, respectively. Therefore, we hypothesized that targeting SULT2B1b could diminish inflammation in macrophages stimulated with ox-LDL, and that miRNAs are involved in this effect. In this study, we found that the proliferative capacity and inflammatory response of macrophages treated with ox-LDL were inhibited by down-regulating the expression of SULT2B1b. Subsequently, upregulation of miR-148a-3p was observed when SULT2B1b expression was silenced. In addition, we further found that miR-148a-3p participates in the improvement of inflammatory response through the miR-148a-3p/IKKβ/IκB/NF-κB signalling pathway. The research revealed the role of miR-148a-3p/IKKβ/IκB/NF-κB axis in the inflammation of macrophages mediated by SULT2BIb, which highlights a promising way to develop novel therapeutic strategies to treat AS by targeting this pathway.

## RESULTS

### Downregulation of SULT2B1b limits proliferation and inflammation in macrophages

**Figure 1:**
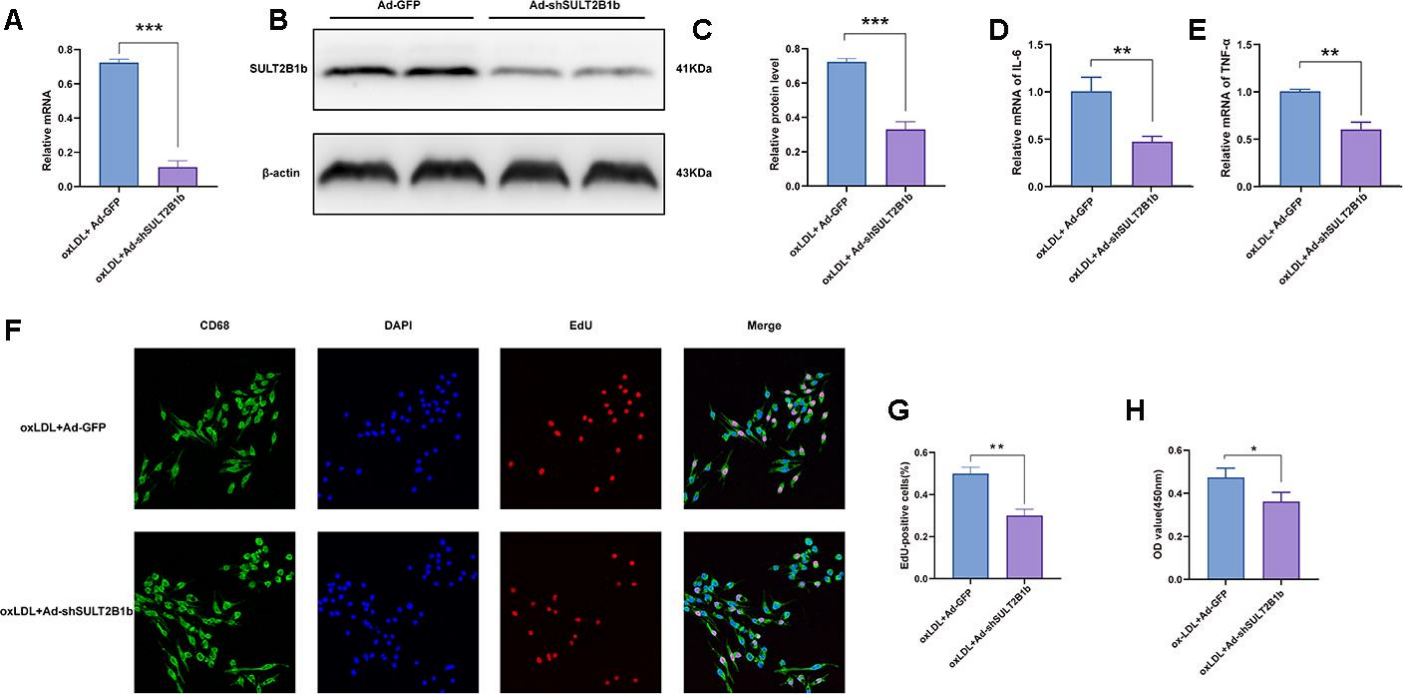
**Downregulation of SULT2B1b limits proliferation and inflammation in macrophages.** (**A**–**C**) Transfection of shSULT2B1b reduces SULT2B1b mRNA and protein levels in macrophages. After 24 hours of adenovirus transfection (MOI=400), RAW264.7 cells were treated with ox-LDL (100 mg/ml) for following 24 hours. (**A**) SULT2B1b mRNA expression was detected by RT-qPCR. Data are represented as the mean±SD (n=3). (**B**, **C**) SULT2B1b protein expression was determined by Western blotting. β-actin was used as a reference control. (**D**, **E**) IL-6 (**D**) and TNF-α (**E**) mRNA expression was determined by RT-qPCR. Data are represented as the mean±SD (n=3). (**F**–**H**) The proliferative ability of RAW264.7 cells was measured by EdU staining and CCK8 assay. Representative images and quantification analysis (**F**, **G**) of the percentage of EdU-positive cells are shown. Nuclei were stained blue with DAPI. Data are shown as the mean±SD (n=3). **p<0.01, ***p<0.001. ns, no significant difference.

To investigate the function of SULT2B1b in macrophages, Raw264.7 macrophages were transfected with Ad-shSULT2B1b-GFP to silence SULT2B1b. To evaluate the transfection efficiency and stability, Ad-GFP and Ad-shSULT2B1b-GFP were transfected into macrophages, respectively. After 48 h of transfection, there was no significant difference between the Ad-GFP- and Ad-shSULT2B1b-GFP-transfected cells (Supplementary Figure 1A, 1B). These data indicated that Ad-shSULT2B1b was efficiently transfected into macrophages. Subsequently, transfection of Ad-shSULT2B1b significantly decreased SULT2B1b mRNA (Figure 1A) and transcriptional (Figure 1B, 1C) levels compared with the Ad-GFP transfection group. Moreover, after 24 h of transfection, we stimulated the macrophages with ox-LDL to induce inflammation response in the macrophages. The mRNA levels of IL-6 and TNF-α in the Ad-shSULT2B1b-GFP transfection group were lower than in the Ad-GFP transfection group (Figure 1D, 1E). These results demonstrated that Ad-shSULT2B1b could significantly decrease SULT2B1b mRNA levels and control inflammation in transfected macrophages. Results of EdU staining (Figure 1F, 1G) and CCK-8 assay (Figure 1H) showed that the proliferative capacity of cells from the ox-LDL+Ad-shSULT2B1b-GFP group was lower than in the ox-LDL+Ad-GFP group, indicating that reducing SULT2B1b expression could impair the proliferative capacity of macrophages. Taken together, these data suggest that SULT2B1b is required for proliferation and inflammation in macrophages after treatment with ox-LDL.

### SULT2B1b impedes miR-148a-3p expression in macrophages

**Figure 2:**
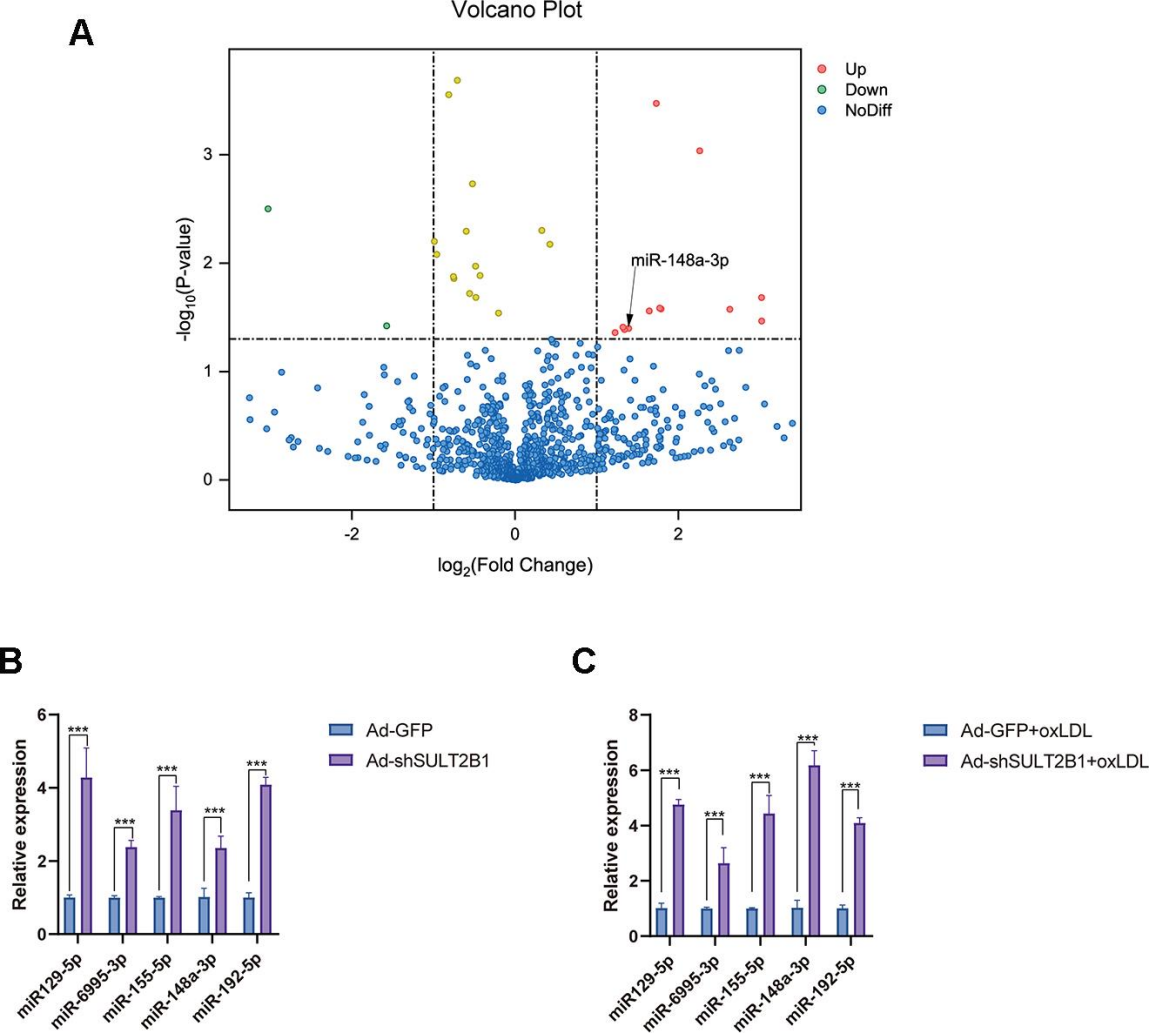
**SULT2B1b impedes miR-148a-3p expression in macrophages.** (**A**) The volcano plot of miRNA profiles represents the significantly up/downregulated miRNAs in RAW264.7 cells transfected with or without Ad-shSULT2B1b. (**B**) Upregulated miRNA expression (miR-129-5p, miR-6995-3p, miR-155-3p, miR-148a-3p and miR-192-5p) was detected by RT-qPCR. (**C**) After transfection with Ad-shSULT2B1b and following stimulation with ox-LDL, miR-148a-3p levels significantly increased among the miRNAs. Data are shown as the mean±SD (n=4). **p<0.01, ***p<0.001. ns, no significant difference.

miRNAs are involved in the development of inflammation and AS progression [22–24]. To determine whether miRNAs play roles in regulating the function of SULT2B1b during inflammation and AS, we performed miRNA-Seq to identify differentially expressed miRNAs between the Ad-GFP- and Ad-shSULT2B1b-GFP-transfected groups. As shown in Figure 2A, we found several differentially expressed miRNAs in these two transfection groups, particularly miR-148a-3p, which was significantly increased after downregulation of SULT2B1b (Figure 2A, 2B). To further investigate whether the effects of SULT2B1b were mediated by regulating the expression of miRNAs in macrophage-related inflammation, we examined the expression profile of miRNAs in macrophages after ox-LDL treatment. The expression of miR-129-5p, miR-6995-3p, miR-155-5p, miR-148a-3p and miR-192-5p was significantly increased in macrophages transfected with Ad-shSULT2B1b upon ox-LDL treatment, whereas Ad-GFP-transfected macrophages did not show these results (Figure 2C). Additionally, previous studies demonstrated that miR-148a-3p was involved in the inflammation response [25, 26]. Taken together, these results indicate that SULT2B1b downregulates miR-148a-3p expression in ox-LDL-treated macrophages, and thus miR-148a-3p could be a downstream target of SULT2B1b in inflammation response.

### miR-148a-3p mediates the regulation of SULT2B1b in macrophages

**Figure 3:**
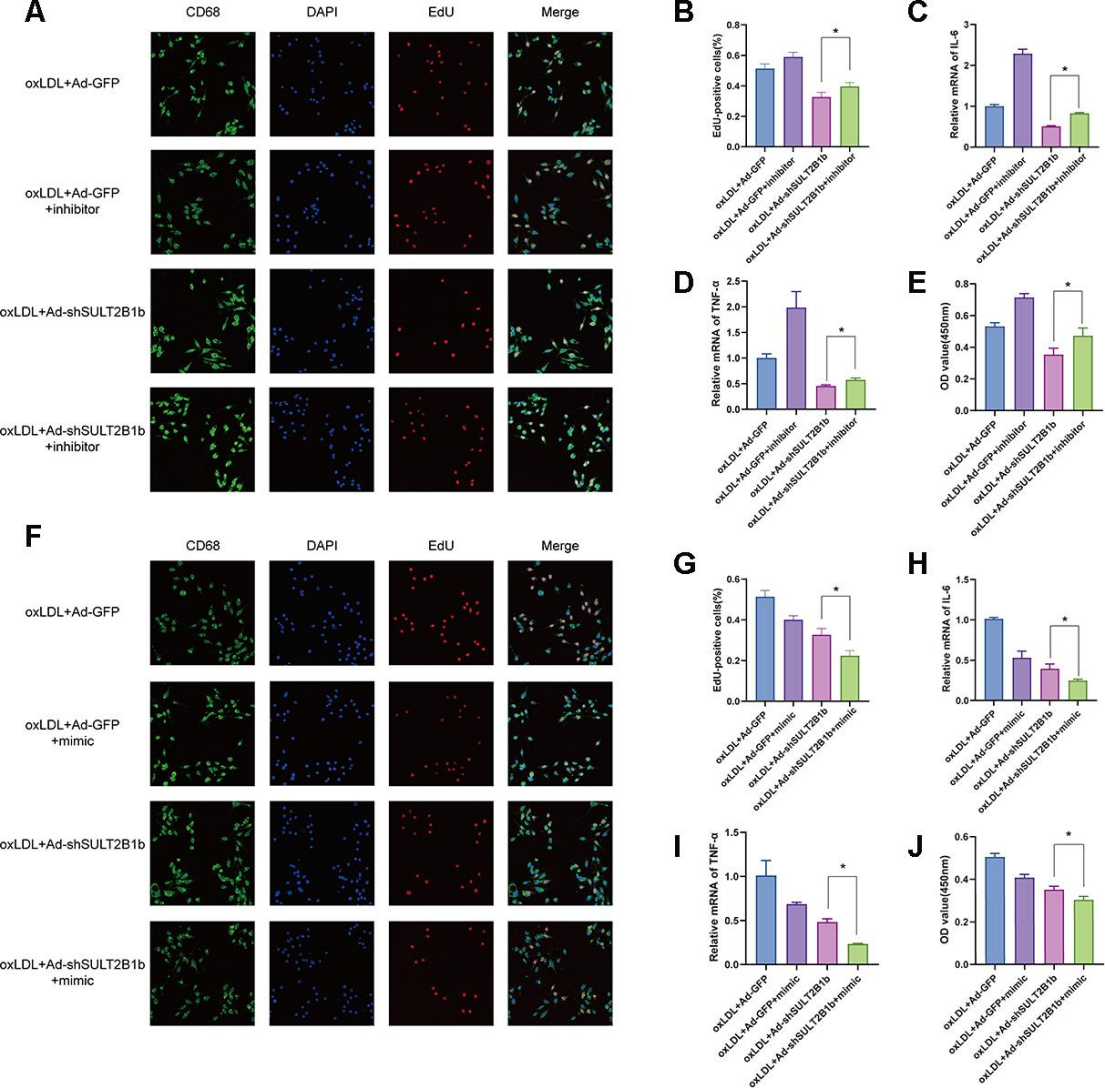
**miR-148a-3p mediates the regulation of SULT2B1b in macrophages.** (**A**–**E**) Following treatment with ox-LDL, Ad-GFP- or Ad-shSULT2B1b-transfected Raw264.7 cells were treated with Ad-GFP or Ad-shSULT2B1b. Subsequently, the cells were transfected with/without miR-148a-3p inhibitor, respectively. (**A**–**C**) The proliferative ability s was detected by EdU staining and CCK-8 assay. (**D**, **E**) IL-6 and TNF-α mRNA levels were determined by RT-PCR. (**F**–**J**) Raw264.7 cells were transfected with Ad-GFP or Ad-shSULT2B1b and the treated with ox-LDL. Then, the cells were transfected with/without miR-148a-3p mimic. (**F**–**H**) The EdU staining and CCK-8 assay data showed the proliferative capacity of the cells. (**I**, **J**) IL-6 and TNF-α mRNA expression was determined by RT-PCR. Data are shown as the mean±SD (n=3). **p<0.01, ***p<0.001. ns, no significant difference.

To further confirm that regulation of SULT2B1b was mediated by miR-148a-3p, miR148a-3p mimics and inhibitors were used to evaluate the effects of miR148a-3p in macrophages. The results showed that miR148a-3p expression was dramatically increased and decreased when treating with a miR148a-3p mimic and inhibitor, respectively (Supplementary Figure 2A, 2B). Subsequently, the macrophages were transfected with Ad-GFP or Ad-shSULT2B1b-GFP and stimulated with ox-LDL. Then, these macrophages were transfected with miR148a-3p inhibitor or mimic, respectively. EdU staining indicated that transfection of miR148a-3p inhibitor could rescue the proliferative ability of macrophages (Figure 3A, 3B). Consistently, CCK8 assay demonstrated that downregulation of miR148a-3p expression by transfection with miR148a-3p inhibitor could restore macrophage expansion (Figure 3E). Meanwhile, the mRNA levels of inflammatory cytokines, including IL-6 and TNF-α, increased after miR148a-3p inhibitor transfection as shown by RT-qPCR (Figure 3C, 3D). In contrast, after transfection with miR148a-3p mimics, the proliferative capacity and expansion of macrophages were inhibited (Figure 3F–3J). Furthermore, IL-6 and TNF-α mRNA expression was clearly decreased (Figure 3H, 3I). Taken together, these results indicate that miR148a-3p mediates the SULT2B1b pathway to limit proliferation and alleviate inflammation in macrophages.

### SULT2B1b regulates the IKKβ/IκB/NF-κB signalling pathway through miR-148a-3p

**Figure 4:**
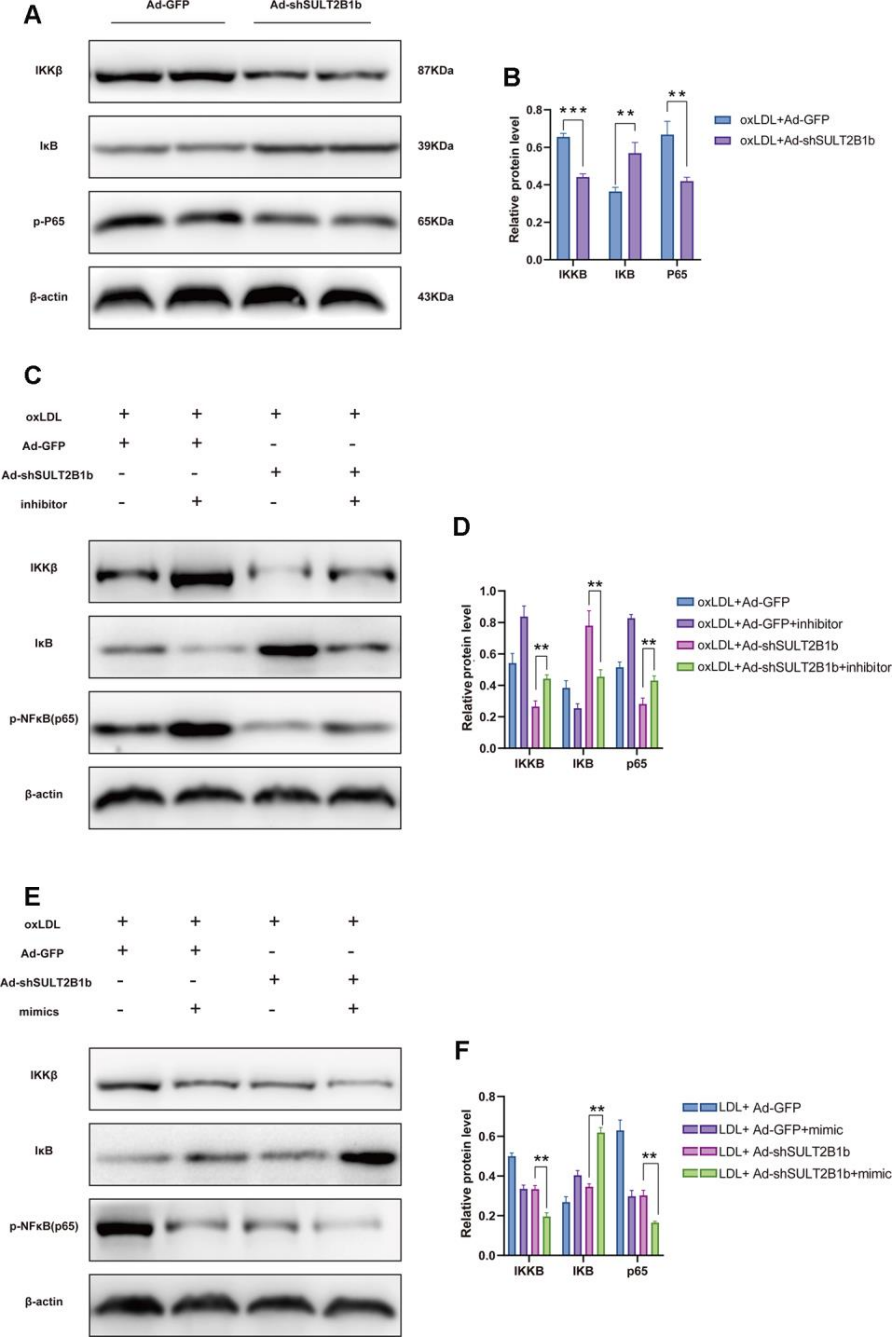
**SULT2B1b regulates the IKKβ/IκB/NF-κB signalling pathway through miR-148a-3p.** (**A**, **B**) The expression levels of IKKβ, IκB and phosphorylated nuclear factor-kappa B (p-p65) protein were determined by Western blotting in Raw264.7 cells transfected with Ad-GFP or Ad-shSULT2B1b. (**C**) Raw264.7 cells were transfected with Ad-GFP or Ad-shSULT2B1b and stimulated with ox-LDL. Then, the cells were transfected with miR-148a-3p inhibitor (**C**, **D**) or miR-148a-3p mimic (**E**, **F**). IKKβ, IκB and p-p65 expression levels were determined by Western blotting (**C**, **E**). β-actin was used as a reference control. The relative levels of IKKβ, IκB and phosphorylated p65 were quantitively compared (**D**, **F**). Data are shown as the mean±SD of at least three independent experiments. **p<0.01, ***p<0.001. ns, no significant difference.

To understand the mechanisms underlying the SULT2B1b-mediated regulation of inflammation in macrophages, IKKβ and IκB expression and nuclear factor-kappa B (p65) phosphorylation levels were further examined. Transfection of Ad-shSULT2B1b-GFP significantly reduced IKKβ and phosphorylated-P65 levels and increased IκB expression levels in ox-LDL-stimulated macrophages, indicating that SULT2B1b mediates the IKKβ/IκB/NF-κB downstream signalling pathway (Figure 4A, 4B). To further investigate whether miR148a-3p is involved in this shSULT2B1b-mediated IKKβ/IκB/P65 signalling pathway, miR148a-3p inhibitors or mimics were transfected into macrophages after Ad-shSULT2B1b transfection and ox-LDL stimulation. The results demonstrated that IKKβ expression and P65 phosphorylation levels were increased after transfection with miR148a-3p inhibitor. Meanwhile, we also found that IκB expression was downregulated in macrophages transfected with miR148a-3p inhibitor (Figure 4C, 4D). Conversely, transfection of miR148a-3p mimics induced the upregulation of IKKβ expression and P65 phosphorylation and clearly decreased IκB expression (Figure 4E, 4F). Taken together, these data suggest that miR148a-3p mediates SULT2B1b regulation of macrophage activities via the IKKβ/IκB/NF-κB signalling pathway.

### The IKKβ mRNA 3'-UTR is a direct target of miR-148a-3p in macrophages

**Figure 5:**
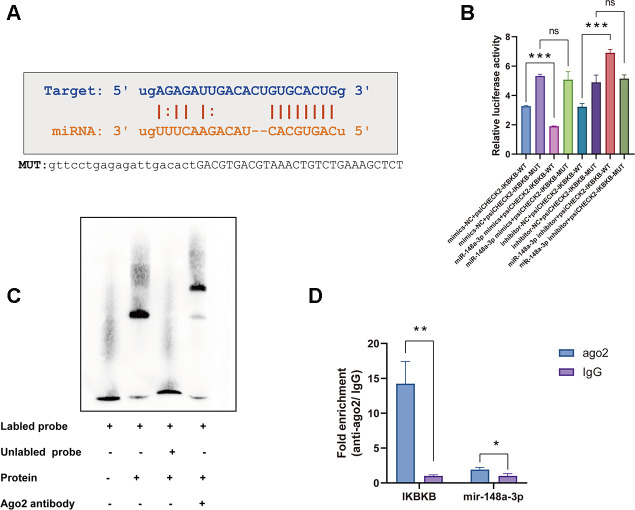
**The IKKβ mRNA 3'-UTR is a direct target of miR-148a-3p in macrophages.** (**A**) The IKKβ mRNA 3'-UTR sequence was aligned with the miR-148a-3p seed sequence. The sequence highlighted with red is potentially recognized. The mutated IKKβ mRNA 3'-UTR sequence is shown. (**B**) RAW264.7 cells were transfected with miR-148a-3p mimic or inhibitor together with a reporter containing wild type IKKβ (psiCHECK2-WT) or mutant IKKβ (psiCHECK2-MUT) as indicated. Data are shown as the relative ratio of firefly luciferase activity to Renilla luciferase activity. (**C**) EMSA for proteins extracted from Raw264.7 cells. (**D**) Co-immunoprecipitation with mouse monoclonal anti-Ago2 or preimmune IgG from extracts of Raw264.7 cells. mRNA levels in immunoprecipitates were determined by RT-qPCR. Data are shown as the mean±SD of at least three independent experiments. **p<0.01, ***p<0.001. ns indicates no significant difference.

Recent studies have reported that miR148a-3p plays a role in inflammation response by targeting different molecules, especially IKKβ [25, 26]. We therefore investigated whether IKKβ is a candidate target of miR148a-3p that is directly involved in macrophage inflammation. We used miRcode and TargetScan to predict miR148a-3p targets and identified one motif in the IKKβ mRNA 3'-UTR that is potentially recognized by miR148a-3p (Figure 5A). To confirm this, we inserted the 3'-UTR sequence of IKKβ mRNA into a plasmid to construct a reporter vector. We found that transfection with miR-148a-3p mimic reduced the fluorescence intensity, whereas transfection with a miR-148a-3p inhibitor increased the fluorescence intensity (Figure 5B). Furthermore, according to the predicted binding region, we constructed an IKKβ mutant plasmid. We found that the fluorescence intensity did not change (Figure 5B) regardless of whether the miR-148a-3p mimic or miR-148a-3p inhibitor was added. In recent studies, it has been found that when a miRNA binds to a mRNA, it mainly binds through an Ago protein [27]. Through an Ago2 RNA immunoprecipitation (RIP) experiment, we found that Ago2 can bind to miR-148a-3p and IKKβ mRNA (Figure 5D). Using luciferase assay and electrophoretic mobility shift assay (EMSA), we confirmed that Ago2 bound to the IKKβ mRNA 3'-UTR sequence in macrophages (Figure 5C). These data indicate that miR-148a-3p forms a complex with Ago2 and binds to the IKKβ mRNA 3'-UTR.

## DISCUSSION

Lipid metabolism and macrophage inflammatory response are two research hotspots in the field of AS, and miRNAs are also considered to play important roles in AS [28, 29]. Growing evidence has demonstrated that SULT2B1b is involved in lipid transport, inflammation and cell proliferation in the liver, brain, lung and skin, mainly by acting as a key enzyme during oxygen sterol vulcanization [30]. This study revealed the role of SULT2B1b in ox-LDL treated Raw264.7 cells and therefore preliminarily evaluated the potential use of SULT2B1b in atherosclerotic therapy. Here, we demonstrated that knockdown of SULT2B1b expression can reduce ox-LDL-treated macrophage proliferation and the expression of inflammatory cytokines, TNF-α and IL-6 included. SULT2B1b promotes its anti-inflammatory and anti-proliferation effects by upregulating miR-148a-3p expression via the IKKβ/IκB/NF-κB pathway, indicating that SULT2B1b is a novel potential target to develop new strategies to treat AS.

Ox-LDL is a recognized traditional and classical risk factor of AS [31]. The biological effects of ox-LDL are mediated partly via NF-κB [32], which can stimulate immune cells to secrete various inflammatory cytokines, such as TNF-α, IL-6, and IL-10 [33, 34]. Under ox-LDL stimulation conditions, monocytes/macrophages transform into foam cells, thus forming atherosclerotic plaques. In this study, we found that ox-LDL significantly promoted macrophage proliferation and increased TNF-α and IL-6 mRNA expression, though these effects are inhibited by knockdown of SULT2B1 expression. Our findings indicate that knocking down SULT2B1b may partially suppress the inflammatory response in ox-LDL-treated macrophages. Therefore, our data show that SULT2B1b knockdown may provide a promising strategy to target inflammation in AS, though the underlying mechanisms still require further elucidation.

To further explore the anti-inflammatory mechanism of SULT2B1b, we measured the expression of miR-148a-3p in Ad-shSULT2B1b and Ad-GFP cells. The data revealed that SULT2B1b knockdown upregulated miR-148a-3p expression. Similar to our findings, Lili Shang et al. found that overexpression of miR-148a-3p could promote endothelial cell proliferation and impair anti-oxidative effects, suggesting that miR-148a-3p might be a potential marker or therapeutic target in AS [35]. Additionally, we also assessed whether miR-148a-3p is a key target through which SULT2B1b regulates the inflammatory response and macrophage proliferation. Using miR-148a-3p analogues and inhibitors, we confirmed that SULT2B1b regulates macrophage proliferation and TNF-α and IL-6 expression through miR-148a-3p. Furthermore, we predicted that IKKβ, encoded by IKBKB, is a target of miR-148a-3p using online informatics tools; this hypothesis was tested and verified by a luciferase reporter assay. MiR-148a-3p attenuated the activation of NF-κB signalling, which is consistent with recent study showing that repression of miR-148a-3p activates NF-κB signalling and increases inflammatory gene expression [26].

NF-κB is an important transcription factor involved in the regulation of cell proliferation, survival and inflammation [36]. It is a dimeric protein composed of Rel family members, namely, P50/p105 (NF-κB1), p52/p100 (NF-κB2), p65 (RelA), RelB and c-Rel [37]. There are many stimuli that can activate NF-κB, including the pro-inflammatory factors IL-6, TNF-α, and integrin 1. These stimuli use different mechanisms to induce NF-κB activation, including the formation of ubiquitin polymers and signalling protein complexes such as the IKK (IB kinase) complex, which is usually composed of the following three major proteins: IKKα, IKKβ and IKKγ. IKKβ can phosphorylate IκB, which combines with the p65 and p50 heterodimers; once phosphorylated, IκB promotes the release and nuclear translocation of active NF-κB [38]. Our results suggest that SULT2B1b knockdown can alleviate inflammation and proliferation in macrophages treated with ox-LDL by upregulating miR-148a-3p expression via the deceased expression of IKKβ and inhibited activation of the NF-κB pathway. Previous studies on SULT2B1b have focused on prostate cancer [39], digestive system tumours [40], lung disease [41], influenza [13], etc. We used lymphocytes to confirm that downregulating SULT2B1b expression can promote cholesterol outflow and inhibit the expression of certain inflammatory factors [15]. In this study, data collected from macrophages show that downregulating SULT2B1b expression can upregulate miRNA expression, thus regulating NF-κB signalling and inhibiting the release of specific inflammatory factors.

**Figure 6:**
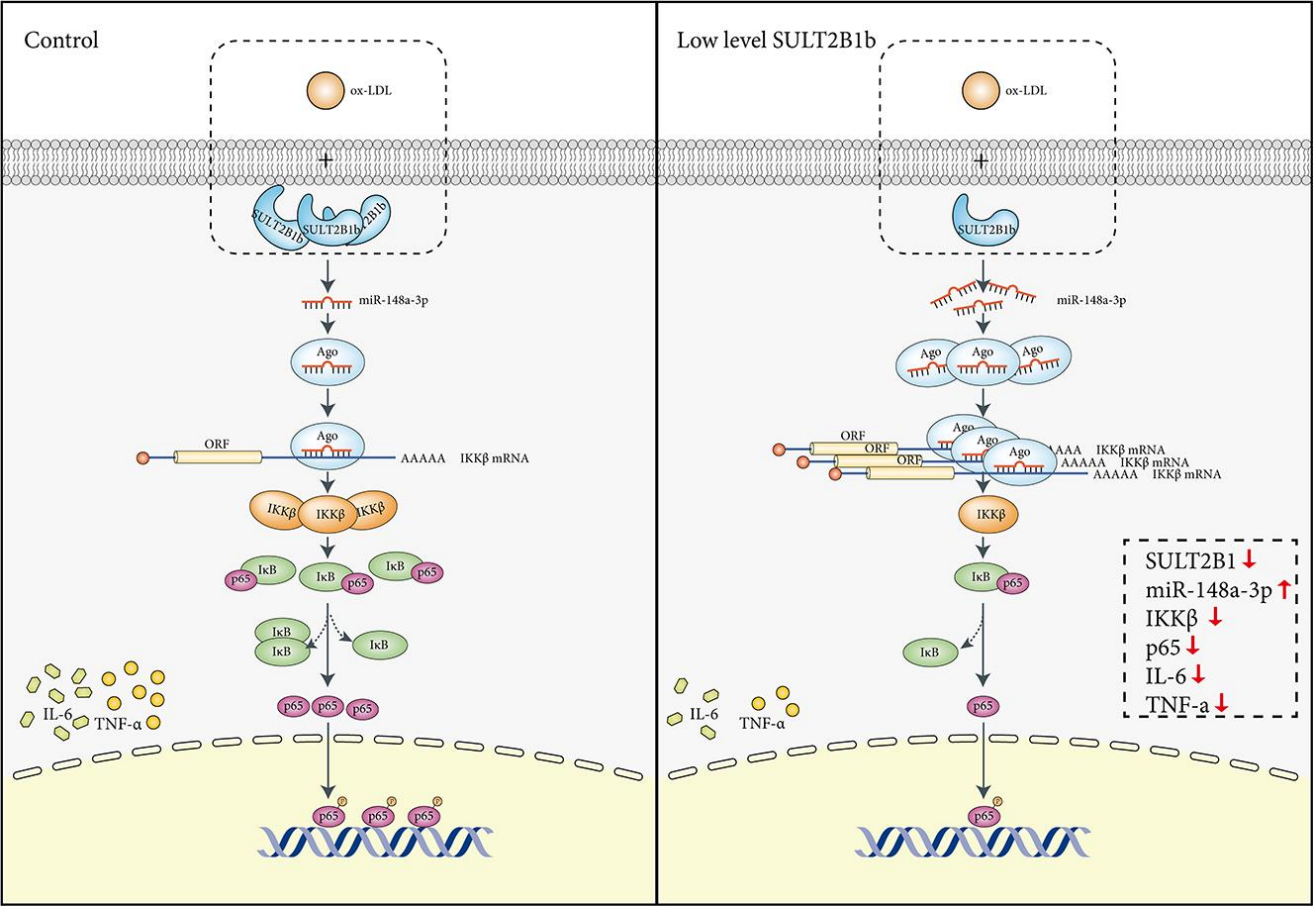
**Mechanisms mediating the role of SULT2B1b in modulating the miR-148a-3p/IKKβ/NF-κB axis in macrophage inflammation.** Stimulation with ox-LDL in Raw264.7 cells can lead to IKKβ/NF-κB pathway activation, thus inducing upregulation of TNF-α and IL-6 expression. In contrast, reduced SULT2B1b expression can increase levels of miR-148a-3p, which can target the IKKβ mRNA 3'-UTR region, resulting in reduction of IKKβ expression and suppress binding of p65 and DNA in the nucleus, which alleviates cellular inflammation and proliferation.

In conclusion, our results suggest for the first time that decreased SULT2B1b expression can reduce the inflammatory response and proliferation of ox-LDL-treated macrophages cells by upregulating miR-148a-3p expression via the IKKβ/IκB/NF-κB signalling pathway (Figure 6). Moreover, these findings reveal the molecular mechanism by which SULT2B1b attenuates the inflammatory response in macrophages treated with ox-LDL. Accordingly, SULT2B1b can be used as an effective target for the prevention and treatment of AS.

## MATERIALS AND METHODS

### Cell culture and treatments

The murine Raw264.7 macrophage (China Centre for Type Culture Collection, CCTCC) were grown at 37° C in a humidified atmosphere of 5% CO_2_ in DMEM containing 10% of foetal bovine serum (FBS). The cells were synchronized by replacing the medium with DMEM supplemented with 0.5% FBS for 12 h before the following experiments. After 12 h, the cells were transfected with adenovirus (Ad)-green fluorescent protein (GFP) or Ad-shSULT2BIb for 48 h and the control group was treated with phosphate-buffered saline (PBS). Subsequently, the macrophages were treated with 100 μg/ml ox-LDL (Human, Yiyuan Biotechnologies, China) in DMEM containing 0.5% FBS for 24 h.

### miRNA sequencing

Total RNA was prepared using TRIZOL (Invitrogen) and the miRNeasy mini kit (QIAGEN) according to the manufacturers’ instructions. The RNA concentrations were quantified using a NanoDrop 2000 spectrophotometer (Thermo Electron Corporation, Beverly, MA, USA) at 260/280 nm. RNA samples were sent to Gminix Biotechnology Co., Ltd. (Shanghai, China) for miRNA deep sequencing on an Illumina HiSeq 2500 sequencing platform with 10 M reads (Illumina, San Diego, CA, USA). Briefly, small RNA fractions consisting of 16 to 26 nucleotides were purified from total RNA and enriched using denaturing polyacrylamide gel electrophoresis (PAGE). Adapters were ligated to the 3’ and 5’ ends using T4 ligase, and small RNAs were subjected to RT-PCR for amplification (12 cycles). The PCR products were further purified using polyacrylamide Tris/borate/EDTA (TBE) gels and then used for sequencing.

### Cell proliferation assay

Cell counting kit-8 (CCK-8) assay was performed to detect the proliferation of Raw264.7 cells. Raw264.7 cells were seeded in 96-well plates at a density of 8×10^3^ cells/well. After stimulation with the pre-designed treatment and adding of CCK-8 solution, the optical density (OD) value of each well was detected at 450 nm using a microplate reader (Spectra Max MD5, Molecular Devices, San Jose, CA, USA).

For the EdU (5-Ethynyl-2’-deoxyuridine) assay, the Raw264.7 cells were incubated with 50 μM EdU solution at 37° C for 2 h using the Cell-Light^TM^ EdU imaging detecting kit (RiboBio, Guangzhou, China) according to the manufacturer's instructions. Finally, EdU-stained cells were analysed with a TCS SP8 confocal laser scanning microscope (LEICA, Germany). The proliferative rate of Raw264.7 cells were expressed as the ratio of EdU-positive cells (red) to total DAPI-positive cells (blue).

### Electrophoretic mobility shift assay (EMSA)

Biotin-labelled oligonucleotides (Supplementary Table 1) of the IKBKB partial 3'-UTR sequence containing the miR-148a-3p binding site were synthesized by Saicheng Biotechnology Co., LTD. The EMSA test was performed with an EMSA/Gel-Shift Kit (Beyotime) according the manufacturer’s instructions. The protein, probes and reaction buffer were mixed in a 20 μl reaction that was incubated at room temperature for 20 minutes. For super shift assays, 0.2 μl of a Ago2 antibody (ab156870; Abcam) was added and incubated with the reaction mixtures for 20 minutes before the addition of labelled probes. After electrophoresed on an 8% polyacrylamide gel electrophoresis (PAGE) gel, the binding products were detected by streptavidin-horseradish peroxidase conjugates according to a SuperSignal West Femto Trial Kit (Pierce, Rockford, IL, USA).

### Quantification of mRNAs and miRNAs by quantitative real-time PCR

RNAiso Plus kit (Takara, Japan) was adopted to extract total RNA of the Raw 264.7 cells. The RNA was reverse-transcribed into cDNA using PrimeScript RT polymerase (Takara, Japan). mRNA-specific cDNA primers were applied to quantitative PCR. The β-actin gene was used as an internal control. Stem-loop reverse transcriptase-PCR (RT-PCR) for mature miRNAs was performed as described previously. The U6 gene was used as an internal control. The RT-qPCR primer sequences are listed in Supplementary Table 2. RT-PCR was performed with the SYBR Green Supermix (Bio-Rad, Hercules, CA, USA). Bulge-loop miRNA primer sets including one RT primer and a pair of qPCR primers were designed by RiboBio (Guangzhou, China). The results are reported as the average ratios of three independent experiments.

### Construction of the luciferase reporter

The sequences of the wild-type or mutant 3′-UTRs of IKKβ containing common miR-148a-3p binding sites were synthesized and cloned downstream of the luciferase reporter gene in the psiCHECK-2 vector (Promega, Madison, WI, USA) using the XhoI and PmeI restriction enzymes. Wild-type or mutated versions of the psiCHECK2-IKKβ-3′-UTR (Promega, Madison, WI, USA) were co-transfected into Raw264.7 cells with either a miR-148a-3p mimic or a miR-148a-3p inhibitor with Lipofectamine 2000 (Invitrogen, Carlsbad, CA, USA). After 24 h, the luciferase activity was determined by using a Dual-Luciferase Reporter Assay System (Promega). The experiment was conducted in triplicate.

### Western blot

After harvested and washed with PBS, the cells were lysed for 30 min in an ice bath, and centrifuged (15 min at 14,000 × g and 4° C), and protein concentrations of cell lysates were quantified by BCA Protein Assay kit(Beyotime, P0010, China). Half of the resulting supernatant was loaded onto a 10% gel for SDS-PAGE and the other half was applied to a 10% gel. Then, the gels were transferred to polyvinyl fluoride membranes. After blocking with 5% milk, the membranes were incubated with protein-specific antibodies (Abs) overnight at 4° C. Finally, the membranes were incubated with an HRP-conjugated secondary Ab (Protein Tech Group, Chicago, IL, USA) and detected using an ECL Western blotting detection kit (Sigma Aldrich). The densitometry of Western blotting was measured using the ImageJ software (National Institutes of Health). The experiment was repeated three times.

### RNA interference and cell transfection

Following RNA interference optimisation, small interfering RNAs targeting the murine SULT2B1b gene were co-transfected with a murine SULT2B1 cDNA plasmid into HEK293T cells via Lipofectamine 2000. The sequence of the small interfering RNA producing optimal mouse SULT2B1b knockdown (Supplementary Table 3) was then cloned into the pLKD-CMV-U6-shRNA vector, an Ad containing a multiple cloning sites for the insertion of short hairpin RNA (shRNA) constructs to be driven by an upstream U6 promoter and a downstream cytomegalovirus (CMV) promoter-GFP cassette. Raw264.7 cells were infected with the Ad-virus or negative control virus for 48 h. RT-PCR and Western blotting were used to determine the effects of RNA interference on SULT2B1b expression.

### Statistical analysis

All of the data are shown as the mean values±standard deviations. Statistical significance was analysed with the Student’s t-test or one-way ANOVA coupled with a Bonferroni-Dunn post-hoc test. All analyses were performed using Statistical Product and Service Solutions (SPSS) (version 13.0; Statistical Product and Service Solutions, Chicago, IL). The test was considered significant if *P*-value <0.05.

## Supplementary Material

Supplementary Figures

Supplementary Tables
