# Supplementary material for: Reduced SULT2B1b expression alleviates ox-LDL-induced inflammation by upregulating miR-148-3P via inhibiting the IKKβ/NF-κB pathway in macrophages
Source: Aging (Albany NY). 2021 Jan 10;13(3):3428–42. doi: 10.18632/aging.202273 (PMC7906218; doi:10.18632/aging.202273)
Supplement: Supplementary Figures [file aging-13-202273-s001.pdf]

SUPPLEMENTARY FIGURES

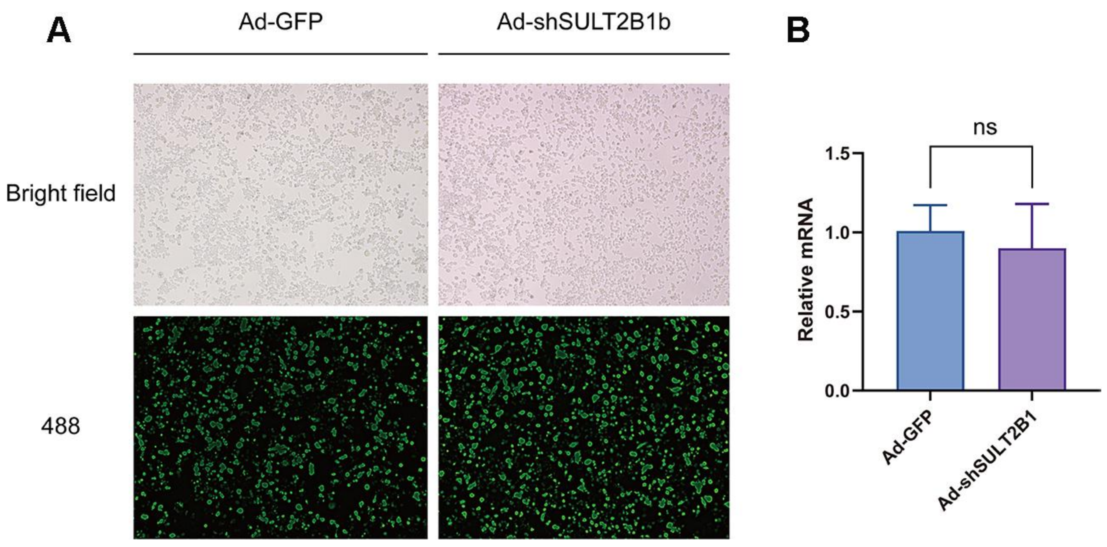

Supplementary Figure 1. Successful transfection of Ad-GFP andAd-shSULT2B1-GFP into macrophages. Raw264.7 cells were transfected with Ad-GFP and Ad-shSULT2B1-GFP, respectively. (**A**) After 24h of transfection, the fluorescent intensity was monitored by inverted fluorescence microscopy. (**B**) The expression of GFP mRNA was determined by RT-qPCR. Data are shown as mean±SD (n=3). **p<0.01, ***p<0.001.

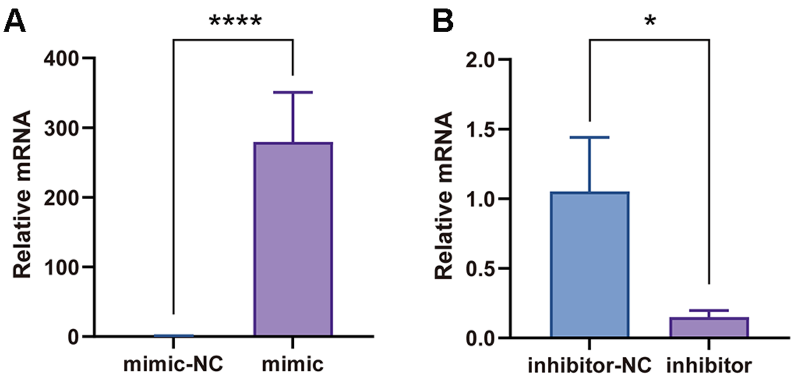

Supplementary Figure 2. The effects of mimic and inhibitor of miR148a-3p on expression level of miR48a-3p in macrophages. (**A**, **B**) The expression of miR148a-3p were significantly increased or decreased after transfection with miR148a-3p mimic (**A**) and inhibitor (**B**), respectively. Data are shown as mean±SD (n=3). **p<0.01, ***p<0.001.
