# Supplementary material for: Reduced SULT2B1b expression alleviates ox-LDL-induced inflammation by upregulating miR-148-3P via inhibiting the IKKβ/NF-κB pathway in macrophages
Source: Aging (Albany NY). 2021 Jan 10;13(3):3428–42. doi: 10.18632/aging.202273 (PMC7906218; doi:10.18632/aging.202273)
Supplement: Supplementary Tables [file aging-13-202273-s002.pdf]

SUPPLEMENTARY TABLES

Supplementary Table

Supplementary Table 1. The probe sequence of biotin-labelled oligonucleotides.

Probe sequence	GAUUGUUCCUGAGAGAUUGACACUGUGCACUGGUAAACUGUC

Supplementary Table 2. Primer sequences for RT-qPCR.

Primer name	Target species	Forward sequence 5′→3′	Reverse sequence 5′→3′
SULT2B1b	mouse	CTGCTTGCCTGTCCTTGGTCAC	AACTGCCTCTCCTGCTGTCCTAC
IL-6	mouse	GCTGACCTCTGGACGCTTAC	CCCATGCCTAACAACTCCAT
TNF-α	mouse	CCACCACGCTCTTCTGTCTA	TGTCCTTCTTGCCCTCCTAA
β-actin	mouse	CAGATCATGTTTGAGACCTTCAAC	TCGAAGTCTAGAGCAACATAGCAC
miR-6995-3p	mouse	TGTGTCCCCTTCCTCTCACAG	CTCTACAGCTATATTGCCAGCC
miR-129-5p	mouse	CTTTTTGCGGTCTGGGCTTG	CTCTACAGCTATATTGCCAGCC
miR-155-5p	mouse	TTAATGCTAATTGTGATAGGGGT	CTCTACAGCTATATTGCCAGCC
miR-192-5p	mouse	AAGCCCTTACCCCAAAAAGTAT	CTCTACAGCTATATTGCCAGCC
miR-148a-3p	mouse	TCAGTGCACTACAGAACTTTGT	CTCTACAGCTATATTGCCAGCC
Reverse transcprition primer		CTCTACAGCTATATTGCCAGCCACACTAATTTTTTTTTTTTTTT	
U6		GTGCTTACTTTAGCAGCACATATATGA	AATATGGAATGCTTCACAAATGC

Supplementary Table 3.The sequence of the small interfering RNA producing optimal mouse SULT2B1b knockdown.

SiSULT2B1b sequence	GGAGGTGAATACTTCAGAT
